# Evaluation of Waste Stabilization Pond Efficiency and Its Effluent Water Quality: a Case Study of Kito Furdisa Campus, Jimma University, Southwest Ethiopia

**DOI:** 10.1155/2022/2800034

**Published:** 2022-05-17

**Authors:** Belay Desye, Biniam Belete, Embay Amare Alemseged, Yonas Angaw, Zinabu Asfaw Gebrezgi

**Affiliations:** ^1^Department of Public Health, College of Medicine and Health Sciences, Adigrat University, Adigrat, Ethiopia; ^2^Department of Public Health, College of Health Sciences, Arsi University, Asella, Ethiopia

## Abstract

Waste stabilization pond (WSP) technology is one of the most promising wastewater treatment methods. In developing countries, including Ethiopia, only a small proportion of the wastewater is being treated. Discharge of untreated wastewater into receiving water bodies may lead to disruption of ecological integrity, economic, and public health risks. However, there is limited evidence on WSP efficiency and effluent water quality in Ethiopia. A laboratory-based cross-sectional study was conducted on 60 wastewater samples. A standard method of procedure was used to collect and analyze samples. SPSS version-24 was used for statistical analysis and a paired *t*-test was used to test for statistical significant differences. A statistically significant difference (*p* < 0.001) in the removal efficiency of BOD_5_ up to 75.3% (117 mg/L effluent) and COD up to 56.5% (457.5 mg/L effluent) was recorded. A statistically significant difference (*p* < 0.001) in the removal efficiency of TN up to 79% (17.4 mg/L effluent), TP up to 69.2% (4.8 mg/L effluent), and PO_4_^−3^ up to 71% (3.36 mg/L effluent) was recorded. Whereas, a statistically significant difference (*p* < 0.001) in the removal efficiency of total coliforms up to 99.99% (3.4 × 10^3^ MPN/100 mL effluent) and fecal coliforms up to 94.3% (8.54 × 10^2^ MPN/100 mL effluent) was recorded. The overall efficiency of the treatment plant was 73.5% and its water quality index of the effluent water quality of WSP was 30. The finding showed that the efficiency of the WSP was judged as satisfactory and the effluent water quality of WSP is unsuitable for the discharge into the environment. Therefore, to improve the efficiency of the WSP and to produce adequately treated water, it required adequate preliminary treatment, modification of the design, desludging of the pond, additional treatment, and frequent monitoring and maintenance of the pond.

## 1. Introduction

Wastewater treatment contributes to the reduction of contamination and pollution of natural waters, and the improvement of aquatic ecosystem health [1]. Water quality is mostly affected by the discharge of poorly treated institutional effluents into surface and ground water sources. The institutional effluents contain organic and inorganic chemicals, biodegradable organic substances, nutrients, and toxic materials [2, 3]. Many institutions discharge their wastewater into receiving water bodies like rivers, streams, lakes, and wetlands without any treatment, which may cause ecological damage and constitute a public health risk that requires proper institutional waste management to mitigate the effects caused by the pollutants [2, 4, 5].

The WSP system is one of the most promising wastewater treatment methods in the world. It is natural, self-sufficient, has a simple design, reducing operator responsibility to manage the system, and a reduction in labor costs. Indeed, WSP is commonly used in many regions around the world, specifically where treating wastewater using conventional treatment methods is costly and in places with year-round mild to warm climate conditions [6, 7]. However, it requires a relatively large area to construct and specific soil condition, and a potential breading sites for mosquitoes. According to the availability of oxygen for the stabilization process, WSP have been classified as anaerobic, facultative, and maturation ponds to achieve effective treatment [8, 9]. Anaerobic and facultative ponds are used for primary and secondary treatment, respectively. They are both designed for the removal of organic matters like biochemical oxygen demand and total suspended solids (TSS). The maturation ponds are used for tertiary treatment of wastewater effluent and designed for pathogens and nutrients removal [10, 11].

WSP provides an impressive method of sustainable wastewater treatment. The effluent of treated wastewater can be reused for irrigation, aquaculture purposes, water conservation, environment, and public health protection. However, the effluent cannot always be reused. Reuse will only be possible if the effluent meets the recommended standards. Many countries have strict regulations for the reuse of treated effluents due to the possibility of the presence of pathogens, mainly for unrestricted irrigation like vegetables that are consumed raw, such as lettuce [12]. Therefore, the operator should monitor the biological and chemical constituents within the system to ensure the properly designed parameters are met with the regulatory treatment efficiency permissible limits [13].

The characteristics of wastewater are essential when determining the efficiency of the treatment plants. WSP treats various waste constituents of nutrients, organics, pathogens, heavy metals, and pharmaceuticals [11]. The major operational parameters for WSP are light penetration, oxygen concentration, temperature, wind, and pond geometry [14]. Factors that can affect the removal efficiency of WSP are raw wastewater strength, organic loading rate, pH, food to microorganism ratio, and hydraulic retention time (HRT) [15].

In developing countries, including Ethiopia, only a small proportion of the wastewater is being treated and the effluent from the WSP system rarely meets the acceptable limit [16, 17]. The poor performance of WSP can be attributed to poor physical and process design, and inadequate operation and maintenance issues [8]. In Ethiopia, many academic institutions, industries, and hospitals discharge their wastewater without maintaining the permissible limit, which can pollute the aquatic ecological system [5, 18]. According to Haddis et al. [2], some of Ethiopian universities are the sources of pollution for communities and to the environment. Despite the presence of an on-site wastewater treatment system in these institutions, the efficiency and overall functionality have been very low. A study conducted in Jimma city, Ethiopia, revealed that there was a lack of proper waste management and low environmental awareness. This results in the indiscriminate discharge of wastewater in the city's waterways, leading to public health risks and environmental impact [4].

Due to the increase in urbanization, higher education institutions, and industrialization in Ethiopia, the wastewater management issue has become the most serious problem recently and will be a major challenge in the future as well [2]. Despite all this, studies on evaluating the efficiency of wastewater treatment technologies used in higher education institutions in Ethiopia are scarce. Therefore, the aim of the present study was to evaluate the WSP efficiency and effluent water quality at Kito Furdisa Campus, Jimma University, Southwest Ethiopia.

## 2. Methods and Materials

### 2.1. Study Area

**Figure 1:**
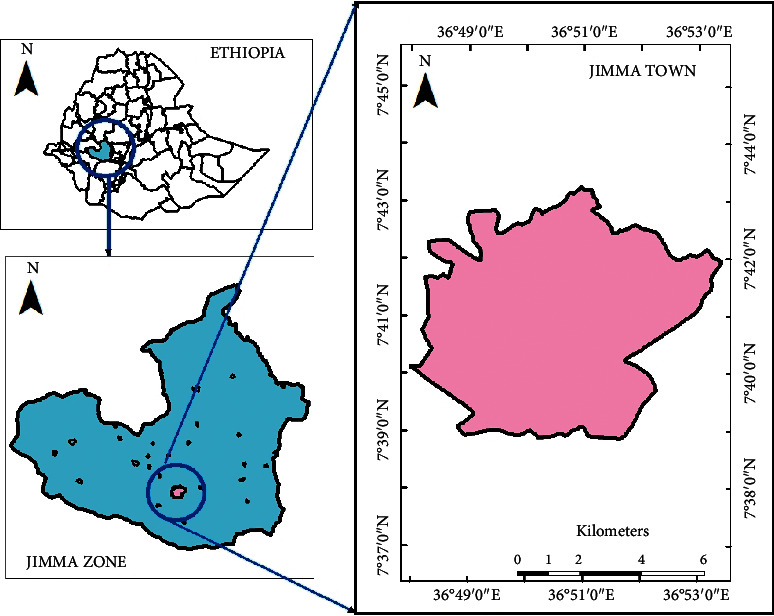
Map of Jimma town, Southwest Ethiopia, 2020.

**Figure 2:**
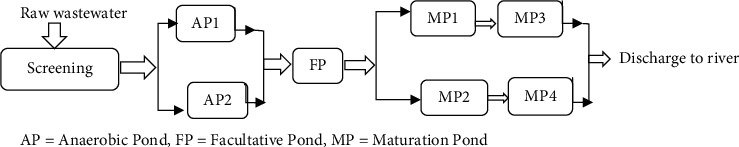
Schematic flow diagram of the WSP in Kito Furdisa Campus, Jimma University, Jimma town, Southwest Ethiopia, 2020.

**Figure 3:**
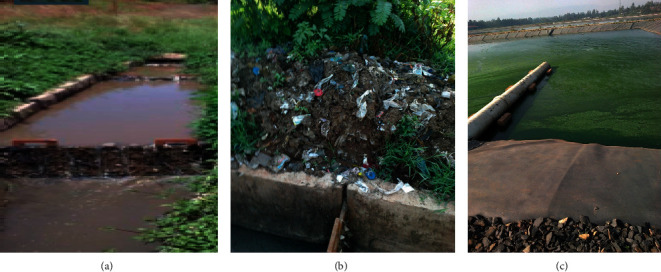
WSP of Kito Furdisa Campus, Jimma University, Jimma town, Southwest Ethiopia, 2020 (photos were taken by the first author).

The study was conducted at Kito Furdisa Campus of Jimma University, Jimma town, 352 Km from the capital city Addis Ababa in the Southwest of Ethiopia as shown in Figure 1. The town of Jimma is found at a latitude and longitude of 7°41′N, 36°50′E. The annual mean temperature of the area is 19.3°C (11.5°C–27.1°C) and the annual rainfall is about 1749.1 mm. The WSP was designed to serve a population of 40,000 and it contains seven ponds as described in Figures 2 and 3.

### 2.2. Study Design and Period

A laboratory-based cross-sectional study was conducted at Kito Furdisa Campus, Jimma University, Southwest Ethiopia, from January 01, 2020 to March 30, 2020.

### 2.3. Experimental Procedure

#### 2.3.1. Wastewater Sample Collection and Laboratory Analysis

Composite samples of untreated wastewater were collected from the influent of the treatment plant. Grab wastewater samples were also collected from influent and effluent of the treatment units during the study period. The sampling period was based on the HRT and samples were taken three times from each sampling location. The HRT of each system during the study period was estimated based on the calculated flow rate and the designed volume of the system.

During the entire study period, a total of 60 wastewater samples were collected and analyzed for the required water quality parameters. The wastewater samples were collected aseptically using 300 milliliter (mL) sterile glass bottles for bacteriological analysis and sterile one-litter polyethylene (PET) bottles for physicochemical analysis. Prior to sampling, the glass bottles were sterilized in an autoclave for 15–20 minutes at 120°C and the PET bottles were washed and rinsed with distilled water. The samples were sealed, labeled, and transported in an icebox (4°C) to the Environmental Health Science and Technology Laboratory, Jimma University. The sampling protocol was carried out scrupulously following the standard methods of the American Public Health Association (APHA) [19].

#### 2.3.2. Wastewater Analysis for Physicochemical Parameters

To determine the efficiency of WSP operation, physicochemical parameters were measured. Physical parameters like temperature (T^o^), pH, electrical conductivity (EC), dissolved oxygen (DO), and turbidity were measured onsite immediately after sampling using a pretested and calibrated portable digital multiparameter probe. Chemical oxidation demand (COD), total nitrogen (TN), ammonia nitrogen (NH_3_-N), nitrate nitrogen (NO_3_-N), total phosphorus (TP), and phosphate (PO_4_^−3^) were measured using a spectrophotometer (DR/2010 HACH, Loveland, USA) according to HACH instructions. Whereas, BOD and TSS were determined using the methods described in the standard method of APHA [19].

#### 2.3.3. Wastewater Analysis for Bacteriological Parameters

Total coliform (TC) and fecal coliform (FC) were determined using the most probable number (MPN) method as explained in standard methods of APHA [19].

### 2.4. Water Quality Index (WQI)

A WQI is a numeric expression used to evaluate surface water for the protection of aquatic life in accordance with specific guidelines and to be easily understood by managers and the public [20, 21]. The calculation of index scores using the Canadian Council of Ministers of the Environment (CCME) WQI method can be obtained by using the following relation [22].

(1) $$\begin{array}{c} \text{WQI}=100-\frac{\sqrt{F_{1}^{2}+F_{2}^{2}+F_{3}^{2}}}{1.732}, \end{array}$$

where

F_1_ (Scope) = Number of variables, whose objectives are not met.

(2) $$\begin{array}{c} F1=\frac{\text{Number of failed parameters}}{\text{Total number of parameters}}*100. \end{array}$$

F_2_ (Frequency) = Number of times by which the objectives are not met.

(3) $$\begin{array}{c} F2=\frac{\text{Number of failed tests}}{\text{Total number of tests}}*100. \end{array}$$

F_3_ (Amplitude) = Amount by which the objectives are not met.

(4) $$\begin{array}{c} \text{excursioni}=\frac{\text{Failed test value }i}{\text{Objective }j}-1,\\ \text{nse}=\frac{\sum_{i=0}^{n}\text{excursion }i}{\text{Number of tests}},\\ F3=\frac{\text{nse}}{0.01\text{nse}+0.01}*100. \end{array}$$

The computed WQI values are classified into five categories as follows as depicted in Table 1.

### 2.5. Data Quality Assurance

To maintain the quality of the data, pretests, instruments for calibration, blank measurements, triplicate analysis, and control media were used. In addition, standard methods of sampling techniques and analysis procedures were used.

### 2.6. Data Management and Analysis

The raw data were coded and entered into a Microsoft Excel spreadsheet. After that, the data were exported to SPSS (version-24) for statistical analysis. The data were analyzed using a paired *t*-test to declare a statistical significant difference between the influent and effluent of the treatment plant in terms of BOD_5_, COD, TSS, TN, NO_3_^−^, NH_3_-N, TP, PO4^−3^, TC, and FC. Mean values, standard deviations, and WQI were also calculated. The overall efficiency of the treatment plant was calculated using the following formula.

(5) Re moval Efficiency%=Ci−Ce  Ci∗100,

where Ci is the influent concentration and Ce is the effluent concentration of pollutants.

## 3. Results

The mean raw wastewater flow rate was 2250 m^3^/d, determined by means of the fill and empty method for about seven days at different times of the day, and estimated using the following formula:

(6) $$\begin{array}{c} \text{Flow rate}\left(\frac{m^{3}³}{d}\right)=\frac{\text{Volume}}{\text{time}}. \end{array}$$

### 3.1. Physicochemical and Bacteriological Analysis

The mean values of pH and DO concentration are increasing from influent to effluent of the pond, while the temperature, turbidity, and EC are decreasing. The characteristics of physical parameters in influent of the treatment plant were pH (7.52), DO (1.56 mg/L), T^o^ (25.3°C), turbidity (345 NTU), and EC (1346.4 *μ*S/cm). Whereas, effluent of the treatment plant pH (8.5), DO (2.12 mg/L), T^o^ (21.9°C), turbidity (122.8 NTU), and EC (850.8 *μ*S/cm) were recorded, as depicted in Table 2.

The removal efficiencies of the treatment plant for BOD_5_ and COD were found to be 75.3% (117 mg/L effluent) and 56.5% (457.5 mg/L effluent), respectively. The removal efficiencies of TN and TP were found at 79% (17.4 mg/L effluent) and 69.2% (4.8 mg/L effluent), respectively. Whereas, the removal efficiencies for TC and FC were found to be 99.99% (3.41 × 10^3^ MPN/100 mL effluent) and 94.3% (8.54 × 10^2^ MPN/100 mL effluent), respectively. BOD_5_, COD, TSS, TN, TP, PO_4_^−3^, TC, and FC were found significant in removal efficiency at *p* < 0.001, whereas, NH_3_-N, and NO_3_^–^ were significant at *p* < 0.01 as depicted in Table 3.

**Figure 4:**
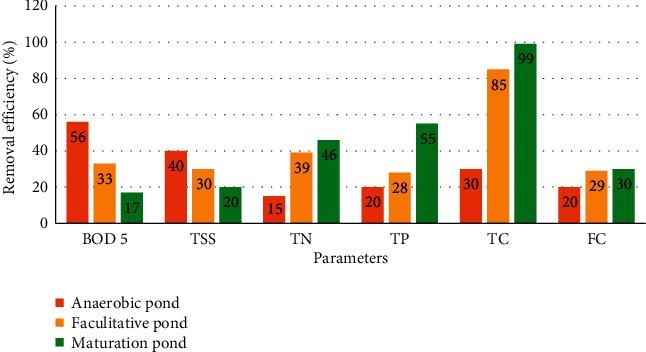
Removal efficiency of anaerobic, facultative, and maturation ponds using selected parameters in Kito Furdisa Campus, Jimma University, Jimma town, Southwest Ethiopia, 2020.

BOD_5_ and TSS were highly removed in the anaerobic (56%, 40%) and facultative ponds (33%, 30%), respectively. Whereas, TN, TP, TC, and FC are highly removed in maturation ponds (46%, 55%, 99%, and 30%), respectively, as depicted in Figure 4.

### 3.2. WQI Calculation

Except pH, T^o^, turbidity, EC, TN, NH_3_-N, and NO_3_^−^, the remaining physicochemical and bacteriological parameters did not meet the permissible limits of the Ethiopian Environmental Protective Authority (EEPA) [23], as depicted in Table 4.

Water quality index (WQI) was calculated using Table 4.

(7) $$\begin{array}{c} F1=\frac{\text{Number of failed parameters}}{\text{Total number of parameters}}*100=\frac{8}{15}*100=53.3,\\ F2=\frac{\text{Number of failed tests}}{\text{Total number of tests}}*100=\frac{40}{75}*100=53.3,\\ F3=\frac{\text{nse}}{0.01\text{nse}+0.01}*100=\frac{22.13}{0.01\left(22.13\right)+0.01}*100=95.7,\\ \text{WQI}=100-\frac{\sqrt{F_{1}^{2}+F_{2}^{2}+F_{3}^{2}}}{1.732}=\;30. \end{array}$$

## 4. Discussion

Anaerobic bacteria are mostly sensitive to pH values of less than 6.2. The pH value was increased from influent (7.52) to effluent (8.5) of the treatment plant. This might be due to increased algal activity in facultative and maturation ponds as CO_2_ is consumed during photosynthesis by algae. The increment in pH might also be due to high ammonia concentrations in the effluent [10, 11]. Similar findings were reported in Gondar, Ethiopia [24], and Hawassa, Ethiopia [25]. The pH value in the effluent of the treatment plant was within the permissible range of EEPA [6–9, 23] and WHO (6.5–8.5) [26].

The temperature value was decreased from influent (25.4°C) to effluent (21.9°C) of the treatment plant. This might be due to the presence of an algal bloom that covers the surface of the pond and blocks the penetration of solar radiation into the bottom of the pond. Furthermore, it might be due to the greater depth and high organic loading of the influent anaerobic pond. As water temperature increases, the solubility of gases like oxygen decreases, with a dramatic effect on organisms inhabiting water bodies [27]. The effluent of treated wastewater temperature was within the permissible limits of EEPA [23]. The current findings were comparable with reported at Woldia University, Ethiopia [28], and Hawassa University, Ethiopia [25].

In the influent of the pond had 1.56 mg/L of O_2_, and the effluent was discharged with 2.12 mg/L of O_2,_ that is, with a concentration higher than the influent. On the reverse, organic matter decreased from 472.9 mg/L of BOD_5_ in the influent and 117 mg/L of BOD_5_ in the effluent due to the consumption of O_2_. The DO concentration of effluent wastewater was far less than the value recommended for aquatic species to respire and perform metabolic activities (≥5 mg/L) [22]. The finding was relatively higher than (0.675 mg/L) reported in Hawassa, Ethiopia [25], and (0.22 mg/L) reported in Sebeta, Ethiopia [29]. The possible reason for this variation might be due to the nature of the raw wastewater, the type of oxidation pond, and the environment [11, 25]. A DO saturation level lower than 5 mg/L can lead to undue stress to the fish and levels reaching below 2 mg/L may result in death. This is an indication that the rate of oxygen production through photosynthesis was lower than the rate of oxygen consumption through respiration and decomposition of organic matter or anaerobic conditions prevailed in the treatment system were unable to enter oxygen into the system through direct diffusion. This fall in DO concentration indicates that the pond is becoming anoxic and some management strategies like aeration with mechanical aerators need to be implemented [10, 11].

A statistically significant difference (*p* < 0.001) in the removal efficiency of BOD_5_ up to 75.3% and COD up to 56.5% was recorded. The findings were somewhat lower than BOD_5_ (76.16%) and COD (67%) reported in Iran [30], even though the removal efficiency depends on the type of oxidation pond and the environment [10, 31]. The BOD_5_ and COD values in the effluent of the pond were 117 mg/L and 457.5 mg/L, respectively. The findings were higher than the limits of EEPA [23] and reported at Hawassa University, Ethiopia [25]. However, the findings were lower than reported in Sebeta, Ethiopia [29]. The variation might be due to the nature of wastewater, the depth, retention time, and environmental factors of the ponds [11].

The higher values of organic loading in the effluent of the pond indicated that the total area of the facultative pond is not sufficient to handle the BOD_5_ concentration of wastewater and a short retention period that should be removed at the preliminary treatment unit [8, 11]. The presence of higher values of BOD_5_ and COD in the treated wastewater may cause depletion of oxygen in receiving water bodies or in the aquatic environment [2, 11]. Therefore, the consequent quality of the effluent and BOD/COD removal depends on the amount of oxygen present, temperature, and retention time of the ponds [32].

Pollutant removal efficiency differences were statistically significant (*p* < 0.001) based on paired *t*-test analysis between influent and effluent units for TN and TP. Removal efficiency of WSP for TN up to 79% (17.4 mg/L effluent) and TP up to 69.2% (4.8 mg/L effluent) was recorded. As Mare et al. [8] describes, a properly functioning WSP can remove 80% of TN and TP. The effluent concentration of TN and TP in the present study was beyond the recommended limits of EEPA [23]. The findings were found lower than reported at Hawassa University, Ethiopia [25]. This discrepancy might be due to the design nature of the pond, the surrounding environmental conditions, and the nature of the raw wastewater. These high values of nitrogen and phosphorus in the effluent of the pond may cause significant pollution in receiving water bodies and other forms of environmental impact [33].

Removal of nitrate up to 70.7% (0.17 mg/L effluent) with a statistically significant difference (*p* < 0.01) and phosphate up to 71% (3.36 mg/L effluent) with a statistically significant (*p* < 0.001) were carried out. The findings of removal efficiency were found higher than reported in Sebeta, Ethiopia [29]. However, the concentration of phosphate in the effluent of the pond was found above the permissible limit of EEPA [23]. Therefore, the presence of high levels of phosphate in the effluent of the pond may cause undesirable phytoplankton growth (eutrophication) in receiving water bodies, which results in algal bloom formation [11, 24].

Although the treatment plant reduced the number of total coliforms (99.99%) and fecal coliforms (94.3%), a statistically significant difference (*p* < 0.001) was the higher reduction, the effluent contains a large number of bacteria. The permissible limit according to WHO [26] for restricted and unrestricted irrigation system is to be 50 MPN/100 mL. However, the effluent of the pond in this study contained 3.41 × 10^3^ MPN/100 mL total coliforms and 8.54 × 10^2^ MPN/100 mL fecal coliforms. The removal efficiency findings were somewhat in consistence with total coliforms (99.74%) and fecal coliforms (99.36%) in Hawassa, Ethiopia [25]. If properly designed and operated, WSP can attain a 99.999% fecal coliform reduction [10].

According to Mara et al. [10] and Engdaw [24], the reduction in the number of colonies depends on pH, retention time, temperature, nutrients, dissolved concentration, and light intensity. From a public health point of view, the presence of pathogens in treated wastewater must be taken into account. There are several problematic pathogens, which can cause various diseases such as cholera, typhoid fever, gastroenteritis, and dysentery [11, 26]. Therefore, alternative options are needed to improve the microbiological quality of effluent wastewater. Mechanical aeration and slow sand filtration with disinfectant of treated wastewater by chlorine may be helpful for better removal [26].

The computed WQI of effluent water quality was 30 and it can be categorized under poor water quality as described by CCME [22]. The finding is supported by [7, 34]. The finding implies that the effluent of the WSP system is inappropriate for discharge to the receiving water bodies and the environment. This might be due to inadequate preliminary treatment to reduce the incoming organic loading, unsuitable design of the pond, and poor maintenance and monitoring system of the treatment plant [31, 35, 36].

### 4.1. Limitations of the Study

This cross-sectional study did not indicate the effect of seasonal variation on the efficiency of the treatment plant and effluent water quality.

## 5. Conclusion

The findings showed that the efficiency of the WSP was judged as satisfactory and the effluent water quality was found to be unsuitable for the discharge into the environment. It discharged wastewater with a high concentration of BOD_5_, COD, TSS, TP, PO_4_^−3^, and coliform bacteria. The treatment plant is still technologically appropriate to treat wastewater, but it needs to upgrade the performance of the pond for better removal and to meet the discharge limit requirements of treated effluent into surface water. To adequately treat wastewater and make it suitable for disposal in the environment, it requires adequate preliminary treatment like septic tank to reduce the incoming organic loading, modification of the design, desludging of the pond, additional treatment, and frequent monitoring and maintenance of the pond.

## Figures and Tables

**Table 1:** Classification of water quality based on the WQI method as described by [22].

WQI value	Status
95–100	Excellent water quality
80–94	Good water quality
60–79	Fair water quality
45–59	Marginal water quality
0–44	Poor water quality

**Table 2:** Mean ± SD of physical parameters of WSP system in Kito Furdisa Campus, Jimma University, Jimma town, Southwest Ethiopia, 2020.

Parameters	Influent concentration	Effluent concentration
pH	7.52 ± 0.08	8.5 ± 0.006
DO (mg/L)	1.56 ± 0.07	2.12 ± 0.08
Temperature (°C)	25.3 ± 0.4	21.9 ± 0.87
Turbidity (NTU)	345 ± 3	122.8 ± 2.35
EC (*μ*S/cm)	1346.4 ± 0.6	850.8 ± 0.09

**Table 3:** Mean ± SD physicochemical and bacteriological quality analysis of WSP system in Kito Furdisa Campus, Jimma University, Jimma town, Southwest Ethiopia, 2020.

Parameters	Influent concentration	Effluent concentration	Removal efficiency (%)
BOD_5_ (mg/L)	472.9 ± 0.42	117 ± 0.64	75.3^*∗∗*^
COD (mg/L)	1051.3 ± 1.6	457.5 ± 2.5	56.5^*∗∗*^
TSS (mg/L)	643.9 ± 1.28	220.5 ± 0.5	65.8^*∗∗*^
TN (mg/L)	82.8 ± 0.66	17.4 ± 0.45	79^*∗∗*^
NH_3_-N (mg/L)	2.5 ± 0.05	1.18 ± 0.04	52.8^*∗*^
NO_3_^−^(mg/L)	0.58 ± 0.03	0.17 ± 0.03	70.7^*∗*^
TP (mg/L)	15.6 ± 0.15	4.8 ± 0.2	69.2^*∗∗*^
PO_4_^−^^3^ (mg/L)	11.6 ± 0.81	3.36 ± 0.13	71^*∗∗*^
Total coliform (MPN/100 mL)	4.87 × 10^8^ ± 0.15	3.41 × 10^3^ ± 0.12	99.99^*∗∗*^
Fecal coliform (MPN/100 mL)	1.5 × 10^4^ ± 0.5	8.54 × 10^2^ ± 0.12	94.3^*∗∗*^

^*∗*^indicates that the parameter was significantly in removal efficiency at *p* < 0.01. ^*∗∗*^indicates that the parameter was significantly in removal efficiency at *p* < 0.001.

**Table 4:** Water quality index of effluent of the WSP system in Kito Furdisa Campus, Jimma University, Jimma town, Southwest Ethiopia, 2020.

Parameters	Number of tests					EEPA [23]
	T1	T2	T3	T4	T5	
pH	8.5	8.5	8.44	8.49	8.56	6–9
DO (mg/L)	2.2^*∗*^	2.05^*∗*^	2.12^*∗*^	2.18^*∗*^	2.15^*∗*^	≥5
Temperature (^O^C)	22.3	20.9	22.5	22.6	21.2	≤40
Turbidity (NTU)	125.2	122.6	120.5	122.7	123	≤300
EC (*μ*S/cm)	850	850.5	850.8	851.2	851.6	≤1000
BOD_5_ (mg/L)	116.7^*∗*^	117.7^*∗*^	116.5^*∗*^	117.1^*∗*^	116.4^*∗*^	≤25
COD (mg/L)	455^*∗*^	460^*∗*^	457.5^*∗*^	461^*∗*^	460.1^*∗*^	≤125
TSS (mg/L)	220^*∗*^	221^*∗*^	220.5^*∗*^	225^*∗*^	220.5^*∗*^	≤50
TN (mg/L)	19.5	16.4	15.5	19.2	16.2	≤20
NH_3_-N (mg/L)	1.15	1.23	1.16	1.23	1.17	≤10
NO_3_^−^ (mg/L)	0.21	0.16	0.15	0.22	0.15	≤45
TP (mg/L)	4.6^*∗*^	4.8^*∗*^	5^*∗*^	6^*∗*^	4.8^*∗*^	≤1
PO4^−3^ (mg/L)	3.5^*∗*^	3.3^*∗*^	3.27^*∗*^	3.6^*∗*^	3.3^*∗*^	≤0.02
Total coliform (MPN/100 mL)	3.5 × 10^3^^*∗*^	3.28 × 10^3^^*∗*^	3.45 × 10^3^^*∗*^	3.56 × 10^3^^*∗*^	3.45 × 10^3^^*∗*^	≤50
Fecal coliform (MPN/100 mL)	8.02 × 10^2^^*∗*^	8.25 × 10^2^^*∗*^	8.15 × 10^2^^*∗*^	8.03 × 10^2^^*∗*^	8.1 × 10^2^^*∗*^	≤10

^*∗*^ = do not meet the guideline EEPA = Ethiopian Environmental Protective Authority.

## Data Availability

The dataset is accessible to the corresponding author upon request.
